# The prospective impact of extradyadic stress on depressive symptoms and the mediating role of intradyadic stress in parents–an actor-partner interdependence mediation model

**DOI:** 10.1371/journal.pone.0311989

**Published:** 2024-11-05

**Authors:** Paula Böhlmann, Judith T. Mack, Victoria Weise, Lara Seefeld, Guy Bodenmann, Anna-Lena Zietlow, Susan Garthus-Niegel

**Affiliations:** 1 Faculty of Medicine, Institute and Policlinic of Occupational and Social Medicine, Technische Universität Dresden, Dresden, Germany; 2 Department of Psychotherapy and Psychosomatic Medicine, Faculty of Medicine and University Hospital Carl Gustav Carus, TUD Dresden University of Technology, Dresden, Germany; 3 Department of Psychology, University of Zurich, Zurich, Switzerland; 4 Faculty of Psychology, Institute of Clinical Psychology and Psychotherapy, Technische Universität Dresden, Dresden, Germany; 5 Faculty of Medicine, Medical School Hamburg, Institute for Systems Medicine (ISM), Hamburg, Germany; 6 Department of Childhood and Families, Norwegian Institute of Public Health, Oslo, Norway; Southwestern University of Finance and Economics, CHINA

## Abstract

**Background:**

Stress outside of the couple relationship (extradyadic stress) can spill over into the couple relationship, increasing stress between the partners (intradyadic stress). Extra- and intradyadic stress are furthermore associated with depressive symptoms. Due to the interdependence of romantic partners, this study aimed to investigate the influence of the person’s own and their partner’s extra- and intradyadic stress on the person’s depressive symptoms in parents of toddlers. The second aim was to evaluate whether intradyadic stress mediates the within-person and between-partner association between extradyadic stress and depressive symptoms.

**Methods:**

Longitudinal data of a community sample of 878 opposite-sex couples, participating in the prospective cohort study DREAM, were collected two and three years after birth. Extra- and intradyadic stress were assessed by the Multidimensional Stress Questionnaire for Couples and depressive symptoms were assessed using the Edinburgh Postnatal Depression Scale. An actor-partner interdependence mediation model was applied to the data, while controlling for the confounder academic degree.

**Results:**

The person’s own extradyadic stress predicted their depressive symptoms one year later, partially mediated by their intradyadic stress. The partner’s extradyadic stress and the person’s own depressive symptoms one year later were only indirectly associated through the person’s own intradyadic stress. In a sensitivity analysis, between-partner effects were no longer significant after including autoregressive pathways.

**Conclusion:**

Our findings highlight the importance of the extradyadic–intradyadic stress spillover for the mental health of women and men with young children. Early targeted interventions could help to prevent later depressive symptoms by reducing stress inside the couple relationship that results from both partners’ stress from outside the couple relationship.

## Introduction

Social relationships, such as couple relationships, are important for a person’s health [1–3]. Being in a couple relationship is associated with better mental health [4] and higher levels of emotional well-being and social support, which in turn are also associated with fewer mental health problems [5]. Furthermore, a longer relationship duration is associated with better physical health [4] and lower rates of depression regardless of whether a person has a history of psychiatric illness [6]. However, being in a couple relationship (especially of low relationship quality) can also negatively affect the health of the partners [2]. There is evidence for stress and emotion transmission between partners, meaning that one partner’s stress can provoke stress in the other partner as well [7]. It is well researched that stress can have an adverse impact on a person’s mental health [8–11], physical health [8, 12], as well as the relationship quality and satisfaction of couples [13–18].

Due to the importance of social relationships to a person’s mental health [1, 7], the role of the romantic partner should be considered when evaluating a person’s mental health. Therefore, understanding the underlying mechanisms (e.g., stress transmission within couple relationships) is important to develop preventive measures and interventions to avoid these adverse consequences.

Speaking of mental health, depressive symptoms are a very common mental health concern, with a lifetime prevalence of diagnosed major depression of 11.6% in Germany [19]. The transition to parenthood is a time of risk for developing depressive symptoms [20]. While perinatal depressive symptoms in mothers are well researched, there is increasing research interest in perinatal depressive symptoms in fathers [21] and in the association between maternal and paternal perinatal depressive symptoms [22]. However, parents of toddlers in general, and fathers in particular, are still an under-researched population. They should not be neglected when studying stress transmission and depressive symptoms, as parents of toddlers face many challenges: For instance, parenting stress is particularly high for parents of toddlers compared to parents of older children [23], and parents have to deal with the autonomy phase of their children [24]. There is evidence that difficulties such as perceived time pressures are not confined to the perinatal period but persist as children grow older [25]. Furthermore, it is common in Germany for mothers to return to work after parental leave during the child’s second year of life [26]. This raises challenges such as balancing work and family [27]. Moreover, having children can also influence the romantic relationship, with children being a major issue in relationship conflicts [28]. During the transition to parenthood, relationship satisfaction has been found to decrease [29, 30], while negative communication and relationship conflict increase [31]. This makes parents of young children an important target group for studying relationship dynamics and stress transmission as well as their impact on mental health.

### Taxonomy for stress in couples

Randall and Bodenmann developed a taxonomy for the description of stress in couples [17]. Three dimensions were postulated: intensity, duration, and origin.

The first dimension–intensity–is classified in major and minor stress. Major stress refers to critical life events, such as the loss of a loved one, severe illness, an accident, unemployment, or adapting to life changes (e.g., birth of a child). Minor stress includes daily stressors related to, for example, children, work setting, or distressing demands of the everyday environment (such as forgetting a meeting or being stuck in a traffic jam) [17, 32].

The second dimension–duration–is divided into acute and chronic stressors [17]. Acute stressors are temporary (e.g., inadequate distribution of chores because of partner’s flu for a few days) and chronic stressors are long-lasting and stable (e.g., inadequate distribution of chores due to partners’ employment situation over several months).

With the third dimension of stress in couples–origin–a distinction is made between stress from outside (extradyadic stress) or inside (intradyadic stress) the couple relationship [17, 33]. Extradyadic stress represents any stressor outside the interaction of the two partners (e.g., low economic status, problems at work, parenting stress, or conflicts with extended family, neighbours, or friends). Intradyadic stress originates within the couple relationship (e.g., conflicts due to different life goals and attitudes, disturbing habits of the partner, or feeling neglected by the partner) [17, 33]. The distinction of extradyadic and intradyadic stress is essential because they have different coping demands [17]. Randall and Bodenmann suggest it would be easier for a partner to show empathy and support in the context of extradyadic stress than in situations of intradyadic stress where one is the cause of the partner’s distress [17].

### Systemic transactional model (STM)

The distinction between extra- and intradyadic stress is based on the systemic transactional model (STM) [34–36]. Central assumptions of the STM are that the two partners in a couple relationship reciprocally influence each other and that stress in one partner can also affect the other partner [34]. Another assumption of the STM is that extradyadic stress can spill over into the couple relationship by increasing the level of intradyadic stress, for example by reducing the time spent together, increasing negative interactions, or encouraging the expression of problematic personality traits [14].

Supporting these hypotheses, evidence exists that the person’s own and the partner’s extradyadic stress are associated with the person’s intradyadic stress [8, 16]. However, another study found this association only between both partners’ critical life events (i.e., major stress) and the person’s intradyadic stress, but not between daily extradyadic minor stress and intradyadic stress [14]. It is important to keep in mind that these findings are based on studies that measured extra- and intradyadic stress at the same time, i.e., cross-sectionally. Therefore, previous studies do not allow causal conclusions to be drawn.

However, the spillover regarding some specific aspects of extra- and intradyadic stress was investigated in longitudinal studies. For instance, parenting stress has been found to impair later relationships quality in parents of toddlers [37]. Diary data suggest that acute extradyadic stress leads to more negative behavior within couples (such as conflict, blame, and withdrawal) [38–41] and chronic extradyadic stress is associated with more negative evaluations of daily relationship experiences [42]. Cooper et al. conclude in their review that “one’s daily experiences of extradyadic stress affect relationship behaviors, thereby creating intradyadic stress that results in a crossover of extradyadic stress from one partner to the other” [43, p. 303].

Examining parents of toddlers would be a valuable contribution to research on dyadic stress and on family health, as it would test the applicability of the STM and the assumption of an extradyadic-intradyadic stress spillover in the context of parenting young children.

### Stress and depressive symptoms–within-person effects

A person’s own stress is associated with their depressive symptoms [44–46]. To evaluate the influence of the extradyadic–intradyadic stress spillover on mental health, these two types of stress need to be considered separately. In terms of extradyadic stress, depressive symptoms are associated with chronic work stress [47–49], work–privacy conflict [50], financial difficulties [51], social isolation from family and friends [52], and parenting stress [53]. Similarly, studies evaluating multiple aspects of extradyadic stress as a whole also found associations with depressive symptoms [8] as well as lower psychological well-being [54]. As noted above, some of these extradyadic stressors are particularly pronounced in parents of toddlers, making them an important target group for investigating the impact of extradyadic stress on depressive symptoms.

In studies examining intradyadic stress, associations with psychiatric disorders (e.g., depression, substance use disorder, anxiety disorders) [55], depressive symptoms [8, 56, 57], and low levels of psychological well-being [54] have been found. A review using research triangulation found evidence that intradyadic stress may be a causal risk factor for depression [58].

Moreover, there is evidence that intradyadic stress partially mediates the association between extradyadic stress and mental health [59]. Therefore, extra- and intradyadic stress do not only predict depressive symptoms independently, but the extradyadic–intradyadic stress spillover proposed by the STM also seems to affect a person’s mental health.

### Stress and depressive symptoms–between-partner effects

Beyond the link between stress and depressive symptoms within a person, which has been highlighted in several previous studies, the romantic partner’s role in the person’s mental health should not be neglected. There is a growing body of research highlighting the importance of evaluating human behavior and cognition beyond the within-person effects using an interpersonal perspective [1, 60] and considering interdependence within couples and families with dyadic analysis methods [61, 62]. In couples, this interdependence between partners can be found, for example, concerning their lifestyle, shared stressors, and their mental and physical health [7, 11]. A higher concordance regarding health and life style of partners is reported in long-term relationships [7, 63]. In terms of partners’ interdependence in depressive symptoms, dyadic evidence was found for a transmission of depressive symptoms from male to female partners over time, mediated by the female partner’s stress appraisal [64], whereas another dyadic study found that the female partner’s depressive symptoms influenced the male partner’s later depressive symptoms [65]. Furthermore, also in young parents, maternal depressive symptoms have been found to predict paternal depressive symptoms in the postpartum period [66, 67].

So far, only few studies conducted dyadic analyses to investigate the association between a person’s extra- or intradyadic stress not only with their own mental health, but also with their partner’s mental health. For instance, the female partner’s stress was associated with the male partner’s depressive symptoms in older rural couples [68]. A study with expectant parents reported no direct association between one romantic partner’s perceived stress and the other partner’s prenatal depressive symptoms for women and men, but an indirect effect between the female partner’s stress and male partner’s prenatal depressive symptoms mediated by the male partner’s relationship satisfaction [46]. In terms of extradyadic stress, there is evidence that the male partner’s critical life events are associated with the female partner’s depressive symptoms [69] and that the female partner’s effort–reward imbalance at work is associated with the male partner’s depressive symptoms [70]. However, another study, investigating the association between minority stress (being stigmatized because of their sexual orientation) and internalizing symptoms in same-sex couples, revealed no effects between the partners [71]. In terms of intradyadic stress, an indirect association between the father’s intradyadic stress and the mother’s prenatal depressive symptoms completely mediated by relationship intimacy was found [56].

These mixed results can be explained in various ways. The studies have focused on different sources of stress (e.g., specific minority stress [71] vs. stress perception in general [46]). Often, stress was also assessed differently, either by scales measuring subjective perceived stress, stress intensity, or the objective presence of specific stressors. Moreover, the studies differed in terms of the investigated sample. It is likely that expectant couples [46, 56, 69], full-time working couples [70], opposite-sex couples older than 50 years [68], and same-sex couples [71] differ in some aspects like type of prominent stressor or relationships dynamics. Therefore, it can be assumed that observed patterns of the influence of the partner’s stress on a person’s own depressive symptoms may not be generalizable to parents of toddlers. Having young children is associated with changes in coping and communication between the partners [31] and extradyadic stressors (such as higher parenting stress [23] or returning to work after parental leave during the child’s second year of life [26], time pressure [25]) are also common in this time. Hence, research on extra- and intradyadic stress and their influence on depressive symptoms is necessary in a sample of parents of toddlers.

To sum up the state of research on the within-person and between-partner associations between stress and depressive symptoms, there is a solid empirical basis for within-person association between the different types of stress and depressive symptoms. However, results are mixed regarding the influence of the partner’s extra- and intradyadic stress on a person’s own depressive symptoms. Direct effects [68, 69], only indirect effects [46, 56], or no effects at all [71] between the partner’s stress and the person’s depressive symptoms were reported. Little is known about potential underlying mechanisms. There is evidence indicating sex differences in vulnerability to the partner’s stress [46, 56, 68–70], but the reported results are inconsistent. Due to the lack of (longitudinal) studies, the low consistency of existing evidence, and the neglected group of parents of toddlers, the between-partner association between stress and depressive symptoms needs further investigation.

### Current study

Considering the theories and research described above, the aim of the current study was to examine the prospective associations between extra- and intradyadic stress and parental depressive symptoms. The study contributes to the existing literature by 1) applying a longitudinal perspective on the extradyadic–intradyadic stress spillover, 2) considering the interdependence in couples regarding their extra- and intradyadic stress and depressive symptoms, 3) testing the mediating role of intradyadic stress for the association between extradyadic stress and depressive symptoms within the person and between the partners, and 4) evaluating possible sex differences in vulnerability to the partner’s stress.

**Fig 1:**
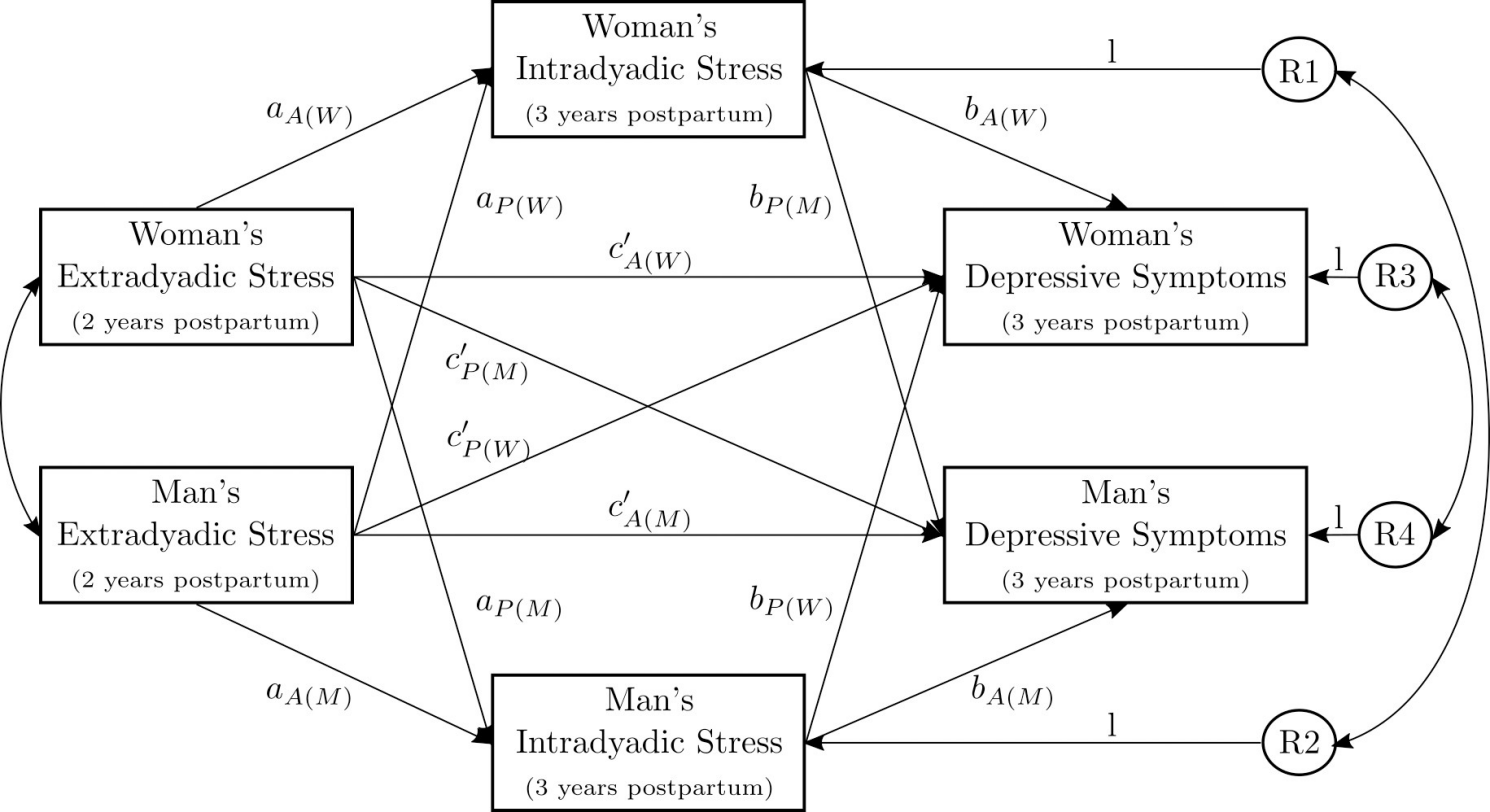
Conceptual model. Actor-partner interdependence mediation model (APIMeM) with chronic extradyadic minor stress as predictor, chronic intradyadic minor stress as mediator, and depressive symptoms as outcome. R1–R4 = residuals of the mediators and outcomes, A(W) = actor effect of women, A(M) = actor effect of men, P(W) = partner effect of women, P(M) = partner effect of men. The model also included confounders and autoregressive paths (not shown in figure).

In terms of the taxonomy of stress in couples [17], only chronic minor stress was considered. Based on the STM [34, 35], the stress spillover’s association with mental health found within the person [59], and the empirical literature on interdependence of partners in stress and mental health [7, 8, 56, 69, 70], an actor-partner interdependence mediation model (APIMeM) [72, 73] was estimated (see Fig 1).

Actor-partner interdependence models (APIMs) have two types of effects—actor and partner effects. Actor effects are within-person associations, for example, the association of the person’s extradyadic stress with the person’s own depressive symptoms. Partner effects are between-partner associations, for example, the association of the partner’s extradyadic stress with the person’s own depressive symptoms.

In terms of actor effects, we hypothesized that a person’s higher extradyadic stress 2 years after birth is prospectively associated with the person’s own higher depressive symptoms one year later and that this association is mediated by the person’s intradyadic stress in women and men. In terms of partner effects, we hypothesized that women’s extradyadic stress 2 years after birth is prospectively associated with men’s depressive symptoms one year later; and men’s extradyadic stress 2 years after birth is prospectively associated with women’s depressive symptoms one year later. Moreover, we investigated whether the association between the partner’s extradyadic stress and the person’s own depressive symptoms is mediated by the person’s own or the partner’s intradyadic stress 3 years after birth in women and men.

## Materials and methods

### Design

Data used in the present study were collected within the Dresden Study on Parenting, Work, and Mental Health (“**DR**esdner Studie zu **E**lternschaft, **A**rbeit und **M**entaler Gesundheit”, **DREAM**). DREAM is a prospective multi-method cohort study that investigates, for example, parental work participation, role distribution, stress factors, and how these are associated with perinatal outcomes and the long-term mental and somatic health of the family [74]. The participants were recruited as a community sample of pregnant women and their partners from Dresden, Germany if they had sufficient German skills. Recruitment took place during pregnancy mainly at birth information evenings in obstetric clinics and in midwife practices from June 13, 2017 to December 31, 2020.

The DREAM study currently consists of six measurement points: T1 during late pregnancy, T2 8 weeks after the anticipated birth date, T3 14 months, T4 2 years, T5 3 years, and T6 4.5 years after birth. At each measurement point, both parents complete a survey comprising different established and validated questionnaires as well as items designed within DREAM. More detailed information about the study is available in the published study protocol [74].

For the present study, data from measurement points T1, T4, and T5 were used, based on data files extracted on March 10, 2023 (version 10 of the quality-assured data files of the DREAM study; prospective data collection ongoing for T4, T5, and T6). On average, participants included in the present study’s analyses completed T1 at gestational week 29.48 (*SD =* 6.46), T4 23.98 months (*SD =* 0.49), and T5 35.96 months (*SD =* 0.54) after birth.

### Sample

The DREAM cohort consists of *N =* 3,860 expectant parents (2,227 mothers, 1,617 male partners, and 16 female partners). Due to the possible influence of sex on the proposed effects [46, 56, 68–70], for this study only data for opposite-sex couples (mothers and male partners) were used. In the following, mothers are referred to as women and male partners as men.

**Fig 2:**
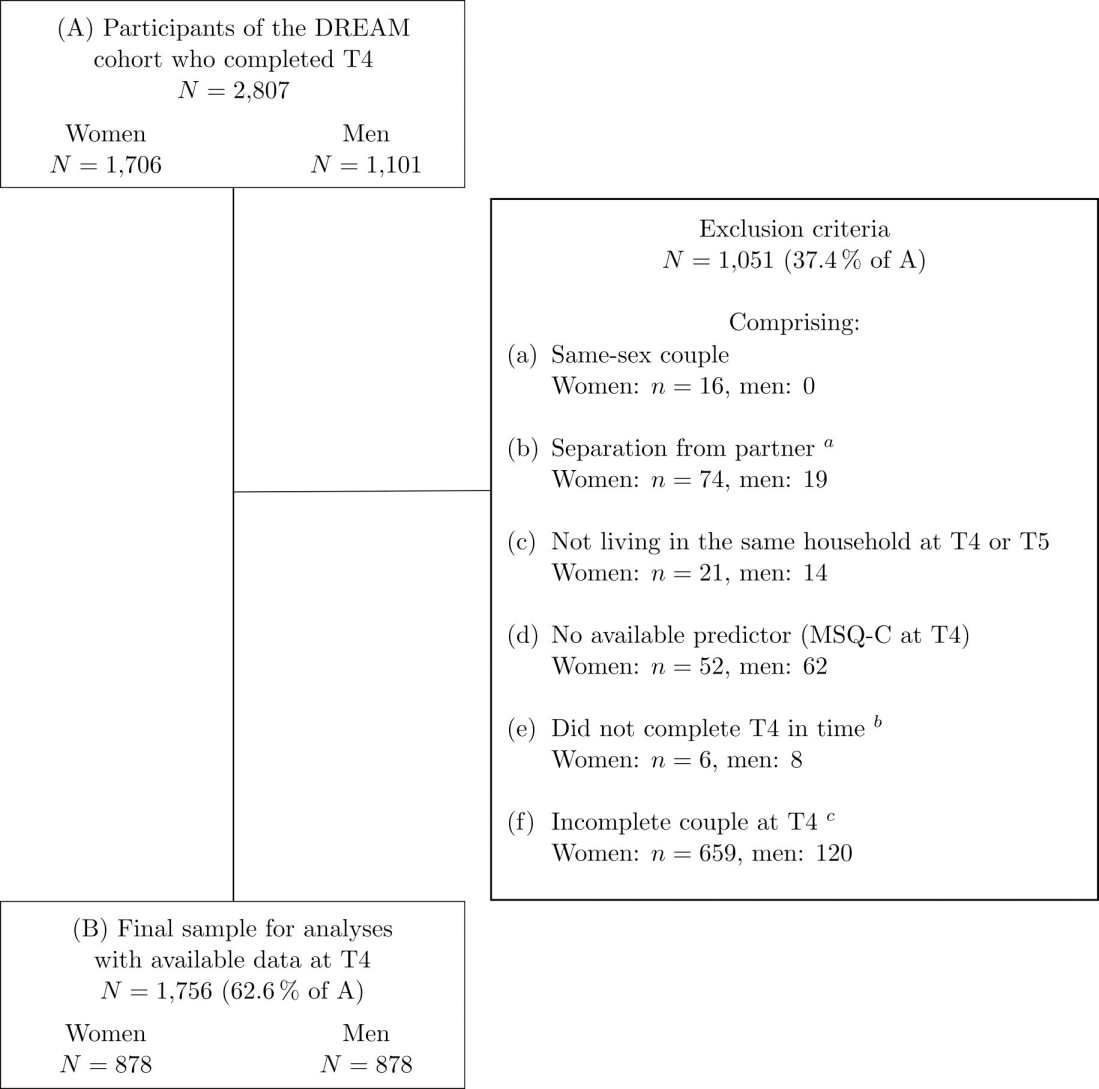
Flowchart of retention rates, attrition, and exclusion criteria. The present sample comprised women and men who completed T4 questionnaires until March 10, 2023 (prospective data collection ongoing). T1 = during pregnancy. T4 = 2 years after birth. T5 = 3 years after birth. MSQ-C = Multidimensional Stress Questionnaire for Couples. ^*a*^ Participants separated from the partner with whom they were in a couple relationship at T1. ^*b*^ Within 2 years ± 2 months after birth. ^*c*^ As only the whole couple was considered at T4, participants were excluded if their partner did not complete T4 questionnaires.

A total of 2,807 women and men, i.e., 73.0% of the initial DREAM cohort, completed the T4 questionnaires (assessment of the predictor variable) and were eligible for the present study. In addition to same-sex couples, participants were excluded from the present study if they had separated from the partner with whom they were in a couple relationship at measurement point T1. For the sake of comparability, participants who did not live permanently with their partner in the same household over the time period considered here (i.e., 10 months before T4 to T5) or did not complete T4 in time (within 2 years ± 2 months after birth) were also excluded. Since the data of the respective partners were combined in the analyses, the whole couple was excluded from the study if at least one of the partners did not meet the inclusion criteria. The final sample for analyses consisted of *N =* 1,756 participants (*n =* 878 women and *n =* 878 men), which are paired in 878 opposite-sex couples. The exclusion process and retention rates are visualized in Fig 2.

Missing data at T5 were estimated via full information maximum likelihood method (FIML); thus, this was not an exclusion criterion. However, to assure comparability, T5 questionnaires were deleted if they were not completed within two months of the due date, i.e., within 3 years ± 2 months after birth. T5 questionnaires were completed by 1,010 participants of the final sample (536 women and 474 men). Therefore, T5 data were estimated for 746 participants.

#### Sample characteristics

The mean age of the participants was 32.21 years (*SD =* 3.81) for women and 34.75 years (*SD =* 5.00) for men. With 59.1% of the women and 56.0% of the men holding an academic degree, the educational level of the sample was higher than the general educational level of the overall German population [75] and Dresden’s population [76]. As shown in Table 1 the majority of participants was born in Germany and were employed at T4. Approximately half of the participating couples were married when they started their participation in the DREAM study.

**Table 1:** Sample characteristics.

	Women		Men	
	*n =* 878		*n =* 878	
Frequencies	Absolute	Relative	Absolute	Relative
	(*n*)^*a*^	(in %^*b*^)	(*n*)^*a*^	(in %^*b*^)
**Country of birth**				
** Germany**	828	94.7	852	97.7
** Other**	46	5.3	20	2.3
**Educational level**				
** ≤ 10years in school**	171	19.5	220	25.3
** >* *10years in school**	707	80.5	650	74.7
**Academic degree**				
** No academic degree**	356	40.7	369	42.9
** Academic degree**	519	59.3	492	57.1
**Employment status**				
** Unemployed**	186	21.2	34	3.9
** Employed** ^***c***^	692	78.8	844	96.1
**Marital status**				
** Unmarried** ^***d***^	433	49.5	430	49.1
** Married**	442	50.5	445	50.9
**COVID-19 pandemic exposure at T4**				
** Before/after pandemic**	124	14.1	123	14.1
** During pandemic**	753	85.9	751	85.9
**COVID-19 pandemic exposure at T5**				
** Before/after pandemic**	36	6.4	32	6.4
** During pandemic**	523	93.6	466	93.6
	***M* (*SD*)**	**Range**	***M* (*SD*)**	**Range**
**Age in years**	32.21 (3.81)	20.00–44.00	34.75 (5.00)	22.00–58.00
**Number of people living in the household**	3.32 (0.61)	3.00–9.00	3.32 (0.61)	3.00–9.00
**Relationship duration in years** ^***e***^	9.27 (3.99)	2.83–25.57	9.29 (4.00)	2.86–25.62
**Extradyadic stress**^***f***^ **at T4**	1.91 (0.47)	1.00–3.75	1.80 (0.42)	1.00–3.71
**Intradyadic stress** ^***g***^				
** At T4**	1.75 (0.54)	1.00–3.70	1.63 (0.46)	1.00–3.40
** At T5**	1.81 (0.54)	1.00–3.70	1.65 (0.47)	1.00–3.40
**Depressive symptoms** ^***h***^				
** At T4**	6.25 (4.77)	0.00–25.00	4.32 (3.92)	0.00–25.00
** At T5**	6.64 (4.98)	0.00–23.00	4.83 (4.24)	0.00–24.00

T4 = 2 years after birth. T5 = 3 years after birth.

^*a*^ Slight variation in absolute frequencies (*n*) due to missing data of some participants.

^*b*^ Valid percentages.

^*c*^ Comprising full-time, part-time, irregular or marginal employment, or being in an apprenticeship.

^*d*^ Comprising never been married before, divorced, widowed by a previous partner, or unclear.

^*e*^ Slight variation between women and men in *M*, *SD*, and range due to differences in completion date between partners.

^*f*^ Mean score of Multidimensional Stress Questionnaire for Couples (MSQ-C) subscale chronic extradyadic minor stress (range 1 to 4).

^*g*^ Mean score of MSQ-C subscale chronic intradyadic minor stress (range 1 to 4).

^*h*^ Sum score of Edinburgh Postnatal Depression Scale (EPDS) (range 0 to 30).

The mean scores for extradyadic stress at T4 and intradyadic stress at T5 were between “not at all” and “slightly”. Although depressive symptoms are analyzed as a continuous variable in this study, the distribution in descriptive categories is reported for a better understanding of the present sample. Among eligible participants at T5, 65 (12.1%) women and 38 (8.0%) men reported minor depressive symptoms and 70 (13.1%) women and 28 (5.9%) men reported symptoms of probable major depression. In comparison to men, women reported significantly higher levels of extradyadic stress (*t*(1,736) = 5.23, *p <* 0.001, *d =* 0.25), intradyadic stress (*t*(997) = 5.09, *p <* 0.001, *d =* 0.32), and depressive symptoms (*t*(1,003) = 6.23, *p <* 0.001, *d =* 0.39).

#### Attrition analyses

Within the first two attrition analyses (separated for women and men), the final sample at T4 was compared to participants who met the inclusion criteria applicable to baseline during pregnancy (T1; being in an opposite-sex relationship, both partners participate in the DREAM study) but did not complete T4 questionnaires. Logistic regression analyses were performed to test whether academic degree, country of birth, employment status, age, and depressive symptoms at T1 predicted the likelihood of attrition at T4. For women, the variance explained by these variables was low, resulting in Nagelkerke’s *R*^2 =^ .027. Only academic degree predicted women’s attrition significantly (*p <* .001). Having an academic degree was associated with an increased likelihood of completing the T4 questionnaires (*OR =* 1.654, 95%-CI [1.261, 2.171]). For men, the explained variance was also low, as shown by Nagelkerke’s *R*^2 =^ .051. Men’s attrition was significantly predicted by their academic degree (*p <* .001) and depressive symptoms at T1 (*p =* .001). Having an academic degree (*OR =* 2.050, 95%-CI [1.624, 2.587]) and low depressive symptoms (*OR =* 0.948, 95%-CI [0.918, 0.980]) increased the likelihood of completing the T4 questionnaires for men.

Next, attrition analyses were performed among participants included in the present study’s final sample. To check for deviation from randomness regarding dropout between T4 and T5, participants who completed T5 questionnaires were compared to those who did not complete T5 questionnaires and whose T5 data were estimated using FIML. The explained variance regarding attrition between T4 and T5 was low for women (Nagelkerke’s *R*^2 =^ .014) and men (Nagelkerke’s *R*^2 =^ .012). Sociodemographic variables (age, academic degree, employment status, relationship duration) and primary variables of the present study at T4 (extradyadic stress, intradyadic stress, depressive symptoms) were not associated with the likelihood of missing data at T5.

### Measures

For all psychometric scales, mean replacement was used to substitute missing values in cases where < 20% of items of the scale were missing.

#### Extradyadic stress

The 8-item subscale chronic extradyadic minor stress of the German version of the Multidimensional Stress Questionnaire for Couples (MSQ-C) [77] was used to assess minor stressors external to the couple relationship. This contains, for example, job-related difficulties, conflicts with the family of origin or other people in the social environment, challenging living or financial situations. A sample item is “Finances (debts, lack of money, no raise, etc.)”. For each item, participants rated how stressful they perceived this aspect of their daily life over the past 10 months using a 4-point Likert scale from 1 = “not at all” to 4 = “highly stressful”. The MSQ-C mean score ranges from 1 to 4. Higher scores indicate higher levels of perceived extradyadic stress. The mean score at T4 (2 years after birth) was used as predictor for the analyses. The internal consistency at T4 (Cronbach’s α = .68 for women and α = .67 for men) was in line with former studies [16]. High internal consistency was not expected due to the heterogeneity in assessed life domains and the multi-dimensional nature of extradyadic stress. Therefore, the score can be used as a measure for a person’s stress level originating from extradyadic sources [8, 78].

#### Intradyadic stress

The 10-item subscale chronic intradyadic minor stress of the German version of the MSQ-C, was used to assess minor stressors within the couple relationship (e.g., conflicts with the partner, disturbing habits of the partner, feeling controlled or neglected by the partner). A sample item is “Difference of opinion with your partner (conflicts, disputations)”. For each item, participants rated how stressful they perceived this aspect of their relationship over the past 12 months using a 4-point Likert scale from 1 = “not at all” to 4 = “highly stressful”. The mean score ranges from 1 to 4. Higher scores indicate higher levels of perceived intradyadic stress. The mean score at T5 (3 years after birth) was used as mediator. To control for the interdependence of different observations of the same variables over time (autoregression), the sum score at T4 was used in a sensitivity analysis. In line with other studies [8, 16], the internal consistency was good (Cronbach’s *α =* .86 − .87 for women and *α =* .84 − .85 for men) at T4 and T5.

#### Depressive symptoms

The German version of the Edinburgh Postnatal Depression Scale (EPDS) [79] was used to assess depressive symptoms within the last seven days. Although the EPDS was developed to screen women for depressive symptoms in the postpartum period [80], there is evidence that the EPDS is a reliable and valid instrument to screen fathers [81, 82], women with toddlers [83] or older children [84], and adults from the general population [85] for depressive symptoms. The EPDS has ten items with a 4-point Likert scale from 0 to 3 for each item. A sample item is “I have felt sad or miserable”. The sum score is ranging from 0 to 30. Higher scores indicate a higher severity of depressive symptoms. EPDS scores of 10 or higher are considered as mild depressive symptoms, scores of 13 or higher indicate a probable major depression [79, 82, 84]. The sum score at T5 was used as outcome, and the sum score at T4 was used for autoregression. In line with former studies [86], the internal consistency was good (Cronbach’s *α =* .87 for women and *α =* .84 − .85 for men) at T4 and T5.

#### Confounder

Based on theoretical considerations and empirical findings, relationship duration [6] and academic degree [87, 88] were taken into account as confounders. Relationship duration in years was calculated as the difference between relationship start and completion date of the T5 survey. The participants’ academic degree (0 = no academic degree, 1 = bachelor’s degree or higher) was included as a dichotomous variable in the analyses. Part of the data collection took place during the COVID-19 pandemic. To account for possible effects on mental health [89, 90], COVID-19 pandemic exposure was also considered as a potential confounder, measured as a dichotomous variable. Participants were grouped in two categories relating to the date of completion of T5, i.e., the date on which the outcome (depressive symptoms) was assessed. Those who completed T5 between March 10, 2020 and January 15, 2023 were assigned to the “during pandemic” group, otherwise they were placed in the “before/after pandemic” group. The “before/after pandemic” group was the reference category in these analyses.

### Data analyses

The preparatory and descriptive analyses were carried out using IBM SPSS Statistics (Version 27) [91]. Descriptive analyses regarding means, standard deviations, and ranges of sociodemographic variables, predictor (extradyadic stress), mediator (intradyadic stress), outcome (depressive symptoms), and confounder variables (relationship duration, academic degree, COVID-19 pandemic exposure) were performed. Bivariate correlations between these variables were computed separately by sex to 1) describe associations between study’s primary variables descriptively, 2) assess the partners’ interdependence regarding primary variables, and 3) test for associations between the proposed confounders and depressive symptoms to further include only those confounders that significantly correlate with the outcome of at least one of the partners in the main analyses.

For the main analyses, actor and partner effects as proposed in Fig 1 were evaluated using an APIMeM [73] with the software Mplus (Version 8) [92]. The APIMeM is based on the structural equation modelling (SEM) method with robust standard errors. Missing values at T5 were estimated using FIML [93].

First, a saturated model, i.e., an APIMeM for distinguishable dyad members (women and men) with all effects freely estimated, was calculated. It consisted of two predictor variables (women’s and men’s extradyadic stress at T4), two mediator variables (women’s and men’s intradyadic stress at T5), and two outcome variables (women’s and men’s depressive symptoms at T5). The model included direct actor and partner effects between extradyadic stress and intradyadic stress as well as between extradyadic stress or intradyadic stress, respectively, and depressive symptoms. Additionally, indirect actor and partner effects between extradyadic stress and depressive symptoms mediated by intradyadic stress were examined. All four mediation pathways were estimated for both women and men resulting in eight indirect effects. Following the recommendations of Zhao et al. [94], mediation was interpreted only considering the indirect effect. Significant total effects between predictor and outcome were not treated as a requirement for mediation [94].

In the second step, analogue paths of women and men were constrained to be equal to generate a more parsimonious constrained model. The constrained model was compared to the saturated model using a *χ*^2^ difference test to accept and further use the constrained model if the models did not differ significantly. The level of significance was set to *p <* .20 in accordance to Kenny & Ledermann [95]. This procedure has already become established in research [96, 97].

In a third model, confounders were added to the previously accepted model if they were significantly correlated with the outcome. Relationship duration [6], academic degree [87, 88], and COVID-19 pandemic exposure [89, 90] were considered as confounding variables because of their association with depressive symptoms in previous studies.

Due to the interdependence of different observations of the same variables in longitudinal research (autocorrelation), controlling for the previous values allows more meaningful statements on the associations between variables [1, 98, 99]. Therefore, autoregression was additionally applied in a fourth model. The autoregressive model was based on the third model, and further included intradyadic stress and depressive symptoms reported at T4. The autoregressive model was used for a sensitivity analysis to additionally investigate the influence of extradyadic stress on intradyadic stress or depressive symptoms, respectively, beyond the former expression of these variables.

The model fit was calculated using the goodness-of-fit indices: *χ*^2^ statistic; root mean square error of approximation (RMSEA) *<* .06; comparative fit index (CFI) *>*.95; and Tucker-Lewis index (TLI) *>*.95 with cut-offs according to Hu & Bentler [100]. The statistical significance of parameters was evaluated using *p*-values (level of significance: *p <* .05) and 95%-confidence intervals (CIs) of unstandardized coefficients (*b*) [96]. The calculation of the CIs was executed using bootstrapping with 5,000 samples [73, 95]. The regression coefficients for effects between metric variables were standardized for better interpretation using the *SDs* of the predictor (*x*) and outcome (*y*) of the subgroup (*i*, women or men):

$$\Delta =b\cdot \frac{{SD}_{x,i}}{{SD}_{y,i}}$$ (1)

For example, Δ = 0.5 indicates that an increase of 1 SD in extradyadic stress (MSQ-C) is associated with an increase of 0.5 SD in depressive symptoms (EPDS).

### Power analyses

According to Ledermann et al. [101], a post-hoc power analysis was conducted by means of Monte-Carlo simulations with 10,000 random samples using the *simsem* package for the R programming language [102]. Assuming small to medium sized correlations in the population of *r =* .17 among predictors (extradyadic stress), mediators (intradyadic stress), and outcomes (depressive symptoms), an APIMeM with the current sample of *N =* 878 dyads had a power of 91.1% or higher to identify significant direct effects among all estimated regression coefficients in the model and a power of 83.1% or higher to identify significant mediation effects. Therefore, the estimated APIMeM was highly powered to detect small to medium sized actor and partner effects as well as mediation mechanisms.

### Ethics statement

All parts of the study were reviewed and approved by the Ethics Committee of the TUD Dresden University of Technology (No: EK 278062015 and EK 104032024). The participants received written information about aims and procedures of the DREAM study and their right to discontinue their participation at any time. Pseudonymization and confidentiality of data were guaranteed. All participants provided written informed consent and participation was voluntary. The participants received no financial compensation for their participation but incentives (e.g., rompers or coloring books) at each measurement point.

## Results

### Correlational analyses

Correlations between extradyadic stress at T4, intradyadic stress at T5, depressive symptoms at T5, and the proposed confounders were calculated (see Table 2). There were moderate correlations between extradyadic stress, intradyadic stress, and depressive symptoms within a person, for both women and men. Between the partners, these variables showed small positive correlations. Partners’ scores for both extradyadic and intradyadic stress were moderately correlated. Further, the partners’ depressive symptoms showed a small correlation.

**Table 2:** Pearson correlations between primary study variables and potential confounders.

	1	2	3	4	5	6
**Within-person** ^***a***^						
**1. Extradyadic stress (T4)**	—	.407***	.440***	.042	.014	.034
**2. Intradyadic stress (T5)**	.423***	—	.422***	.030	−.078	−.042
**3. Depressive symptoms (T5)**	.442***	.387***	—	−.017	−.103*	.039
**4. Relationship duration (T5)**	.061	.032	−.003	—	.036	−.042
**5. Academic degree (T1)**	−.015	−.048	.018	.049	—	−.059
**6. COVID-19 exp (T5)**	.004	.012	.004	.002	−.011	—
**Between-partner** ^***b***^						
**1. Extradyadic stress (T4)**	.303***	.210***	.158***	.075	.028	.009
**2. Intradyadic stress (T5)**	.187***	.453***	.194***	.038	−.084	.004
**3. Depressive symptoms (T5)**	.136**	.200***	.179***	−.011	−.026	.014
**4. Relationship duration (T5)**	.037	.032	−.019	1.000***	.027	−.030
**5. Academic degree (T1)**	.007	−.057	−.001	.070	.374***	−.043
**6. COVID-19 exp (T5)**	.019	−.041	.034	.003	−.028	.913***

T1 = during pregnancy. T4 = 2 years after birth. T5 = 3 years after birth. COVID-19 exp = COVID-19 pandemic exposure

^*a*^ Within-person correlations of women above and of men below the main diagonal.

^*b*^ Women’s variables in columns and men’s variables in rows.

* *p <* .05.

** *p <* .01.

*** *p <* .001.

The only significant association between depressive symptoms and the proposed confounders was found for academic degree in women. Therefore, academic degree, but not relationship duration and COVID-19 exposure was included in the main analyses. Although academic degree was only correlated with women’s depressive symptoms, women’s and men’s academic degrees were included in the main analyses to keep the number of variables the same for both sexes.

### Main analyses

To test the prior postulated hypotheses, an APIMeM was computed (see Fig 1).

#### Model selection

A χ^2^ difference test was used to decide whether to continue using the saturated model or the constrained model, as described above. As there were no significant differences to the saturated model (*p =* .893), a constrained model with all analogue pathways set equal for women and men was accepted (model fit: *χ*^2 =^ 2.273, *df =* 6, *p =* .893, RMSEA = 0.000, CFI = 1.000, TLI = 1.000). Therefore, effects were assumed to be independent of sex.

In the next step, academic degree was included in the model because of its significant correlation with the outcome (depressive symptoms). Analogue pathways between academic degree and intradyadic stress or depressive symptoms were not set equal for women and men because the model would significantly differ from a model with no restriction on these pathways (*p =* .042). Thus, women and men differed regarding the confounding influence of academic degree.

The resulting model with the included confounder (academic degree) fitted the data well, (*χ*^2 =^ 8.948, *df =* 10, *p =* .537, RMSEA = 0.000, CFI = 1.000, TLI = 1.000). The effects from the model with primary variables (extradyadic stress, intradyadic stress, and depressive symptoms) and the correlating confounder (academic degree) are given and discussed below. For the estimated regression coefficients of the other models, see S1 and S2 Tables.

#### Actor effects

**Fig 3:**
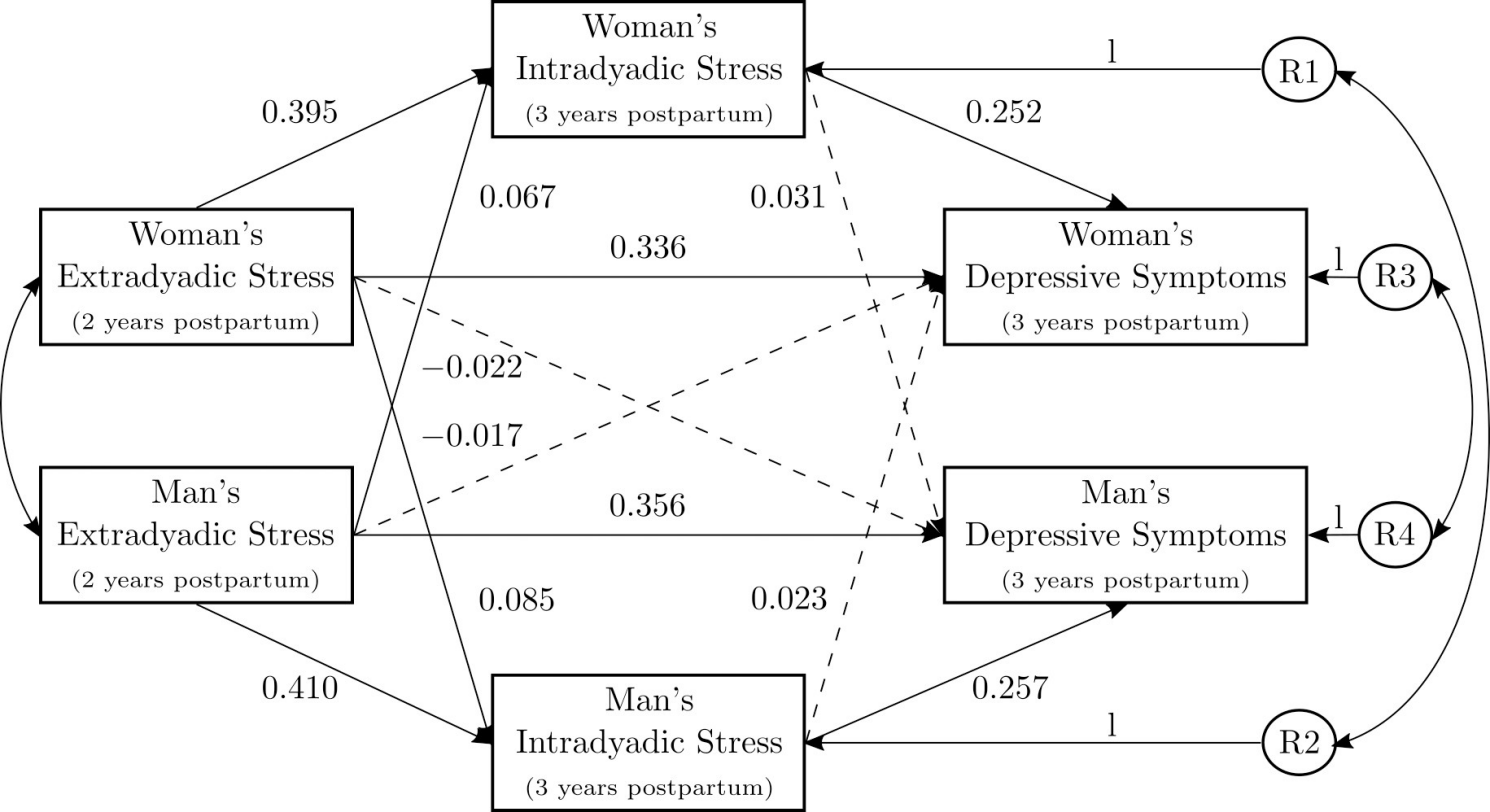
Standardized coefficients (Δ) of direct effects in the actor-partner interdependence mediation model (APIMeM) with included confounder (academic degree). APIMeM with chronic extradyadic minor stress as predictor, chronic intradyadic minor stress as mediator, and depressive symptoms as outcome. R1–R4 = residuals of the mediators and outcomes. The model also included the confounder academic degree (not shown in figure). Solid lines indicate significant paths (*p <* .05). Dashed lines indicate non-significant paths. *χ*^2 =^ 8.948 (*df =* 10, *p =* .537). RMSEA = 0.000. CFI = 1.000. TLI = 1.000.

As shown in Fig 3, all three direct actor pathways between primary variables were significant with small to medium sized effects for both women and men (for tabulated results, see S3 Table). Within a person, extradyadic stress was positively associated with later intradyadic stress (*b =* 0.453, 95%-CI [0.389, 0.519], Δ_♀/♂ *=*_ 0.395*/*0.410, *p <* .001) and intradyadic stress was positively associated with depressive symptoms (*b =* 2.338, 95%-CI [1.660, 3.017], Δ_♀/♂ *=*_ 0.252*/*0.257, *p <* .001). Higher extradyadic stress predicted higher depressive symptoms one year later (*b =* 3.582, 95%-CI [2.901, 4.282], Δ_♀*/*♂_ = 0.336*/*0.356, *p <* .001).

In order to test the mediating role of intradyadic stress, indirect effects were estimated (see Table 3). The association between a person’s extradyadic stress and their own depressive symptoms one year later was partially mediated by their own intradyadic stress (*b =* 1.060, 95%-CI [0.736, 1.419], Δ_♀/♂ *=*_ 0.100*/*0.105, *p <* .001). The significant specific indirect effect accounted for 22.7% of the total effect.

**Table 3:** Indirect effects in the actor-partner interdependence mediation model (APIMeM) with included confounder (academic degree).

	*b*	Δ_♀/♂_	*SE*	*p*	95%-CI	
					Lower	Upper
**ES**_**A**_ **→ DS**_**A**_						
** Total effect**	4.663	0.438/0.464	0.312	< .001	4.052	5.274
** Direct**	3.582	0.336/0.356	0.355	< .001	2.901	4.282
** Total indirect**	1.081	0.102/0.108	0.166	< .001	0.772	1.421
** Specific indirect via IS**_**A**_	1.060	0.100/0.105	0.175	< .001	0.736	1.419
**Specific indirect via IS**_**P**_	0.021	0.002/0.002	0.033	.530	−0.038	0.095
**ES**_**P**_ **→ DS**_**A**_						
** Total effect**	0.113	0.010/0.012	0.314	.719	−0.484	0.736
** Direct**	−0.196	−0.017/−0.022	0.360	.587	−0.893	0.512
** Total indirect**	0.309	0.026/0.034	0.167	.064	−0.020	0.639
** Specific indirect via IS**_**A**_	0.198	0.017/0.022	0.078	.011	0.050	0.364
** Specific indirect via IS**_**P**_	0.111	0.009/0.012	0.158	.483	−0.198	0.427

*b =* unstandardized coefficients. Δ = standardized coefficients separated by sex. *SE =* standard errors of *b*. Two-tailed *p*-values (*p <* .05 in bold). Bootstrapped 95%-CIs (5,000 iterations). A = actor.

P = partner.

*χ*^2 =^ 8.948 (*df =* 10, *p =* .537). RMSEA = 0.000. CFI = 1.000. TLI = 1.000.

#### Partner effects

In terms of partner effects, one of three direct pathways was significant (see Fig 3). The partner’s higher extradyadic stress predicted the person’s higher intradyadic stress one year later, albeit with very small effect sizes (*b =* 0.085, 95%-CI [0.022, 0.147], Δ_♀/♂ *=*_ 0.067*/*0.085, *p =* .007). There were no significant direct partner effects between extradyadic stress and depressive symptoms one year later (*b =* −0.196, 95%-CI [−0.893, 0.512], Δ_♀/♂ *=*_ −0.017*/* − 0.022, *p =* .587) or intradyadic stress and depressive symptoms (*b =* 0.245, 95%-CI [−0.424, 0.930], Δ_♀/♂ *=*_ 0.023*/*0.031, *p =* .481).

Although no significant direct partner effect was found between extradyadic stress and later depressive symptoms, a very small indirect effect was found between the partner’s extradyadic stress and the person’s depressive symptoms mediated by the person’s own intradyadic stress (*b =* 0.198, 95% CI [0.050, 0.364], Δ_♀/♂ *=*_ 0.017*/*0.022, *p =* .011). The partner’s intradyadic stress was not a mediator between the partner’s extradyadic stress and the person’s depressive symptoms (*b =* 0.111, 95%-CI [−0.198, 0.427], Δ_♀*/*♂_ = 0.009*/*0.012, *p =* .483).

#### Confounding influence of academic degree

Holding an academic degree was associated with less depressive symptoms in women (*b =* −0.912, 95%-CI [−0.679, −0.152], *p =* .019). All other associations between academic degree and primary variables were not significant. There were no changes regarding statistical significance or clinically relevant changes in effect sizes of pathways between primary variables after including academic degree (see S3 and S4 Tables in comparison).

#### Results when controlling for autoregression

In a sensitivity analysis, the APIMeM was complemented by intradyadic stress and depressive symptoms at T4 (i.e., the time extradyadic stress was measured). There were no significant differences between the model with autoregressive pathways separated by sex and the model with analogue autoregressive pathways set equal for women and men (*p =* .645). Therefore, the more parsimonious model was used. The model results are shown in S4 Table.

In terms of actor effects, intradyadic stress at T4 or depressive symptoms at T4, respectively, were autocorrelated with the variables one year later at T5 for both women and men. In terms of partner effects, the partner’s higher intradyadic stress at T4 predicted the person’s higher intradyadic stress one year later, but the partner’s depressive symptoms at T4 were not predictive for the person’s depressive symptoms one year later for both women and men.

The previously reported results (see actor effects and partner effects) changed after inclusion of the autoregressive pathways. All direct and indirect actor effects remained significant but decreased in their effect size. The direct partner effect between the partner’s extradyadic stress at T4 and the person’s intradyadic stress at T5 as well as the indirect effect between partner’s extradyadic stress at T4 and the person’s depressive symptoms at T5 mediated through the person’s intradyadic stress at T5 were no longer significant after inclusion of the autoregressive pathways.

## Discussion

This study aimed to investigate the mediating role of intradyadic stress 3 years after birth in the prospective association between extradyadic stress 2 years after birth and depressive symptoms 3 years after birth in parents of young children. We applied an APIMeM under the assumption of interdependence of romantic partners in their stress and depressive symptoms. Within a person, extradyadic stress predicted depressive symptoms one year later and this association was partially mediated by intradyadic stress in both women and men. Between the partners, the partner’s extradyadic stress predicted the person’s intradyadic stress one year later in both women and men. There was no direct association between the partner’s extra- or intradyadic stress and the person’s depressive symptoms in neither women nor men. The partner’s extradyadic stress predicted the person’s depressive symptoms only indirectly, fully mediated by the person’s own intradyadic stress. Sex differences were not found, neither within-person (actor effects) nor between-partners (partner effects).

### Stress and depressive symptoms–within-person effects

Both chronic extra- and intradyadic stress were positively associated with depressive symptoms. This is in accordance with other studies evaluating the association between depressive symptoms and stress from work [47–50], finances [51], children [53], or the romantic partner [8, 55–57]. Thus, our study contributes to the prior empirical evidence by reporting the association between the person’s own chronic stress and depressive symptoms [44].

The association between extradyadic stress 2 years after birth and depressive symptoms 3 years after birth was partially mediated by intradyadic stress 3 years after birth for both women and men, in line with findings reported by Hassan-Abbas [59]. Hence, the extradyadic–intradyadic stress spillover–which was also found by previous studies [8, 14–16, 59, 103]–might impact a person’s depressive symptoms. Extradyadic stress seems to increase depressive symptoms not only directly, but also indirectly by disturbing the couple relationship due to, for example, an increased number of conflicts [38] or the person’s greater awareness of relationship difficulties [42]. The person may perceive these conflicts and relationship difficulties as intradyadic stress, which in turn increases depressive symptoms [8, 55–57].

The completed sensitivity analysis included autoregressive pathways to compensate for autocorrelation of the same variable over time. Thus, we were able to describe changes in intradyadic stress and depressive symptoms explained by extradyadic stress beyond the initial levels of intradyadic stress and depressive symptoms. The associations remained significant in the sensitivity analysis. This provides strong evidence for the extradyadic–intradyadic stress spillover negatively and prospectively affecting the mental health of women and men. Parents of toddlers face many work and parenting challenges that can be distressing. Parents’ own experiences of extradyadic stress can impair the couple relationship and increase their own depressive symptoms.

### Stress and depressive symptoms–between-partner effects

The reported extradyadic–intradyadic stress spillover was not only revealed within a person, but also between the partners. Higher levels of a partner’s extradyadic stress 2 years after birth increased the person’s intradyadic stress one year later. This is in accordance with former studies [8, 16, 103], stating that, for instance, the struggle of the partner with work, family of origin may increase the person’s own perceived stress within the relationship due to, for example, conflicts, lack of intimacy, or unsatisfactory distribution of tasks regarding childcare and household chores. Contrary to the aforementioned studies measuring extra- and intradyadic stress cross-sectionally, the present study provides evidence for a long-term increase of intradyadic stress due to the stress spillover. Our findings support the assumption of the STM that romantic partners reciprocally influence each other in their stress [34] and that stress from outside the couple relationship disturbs the relationship by increasing both partners’ stress inside the couple relationship [14]. Hence, the present study provides evidence that the assumptions of the STM are applicable to parents of toddlers. However, since the actor effect between extradyadic stress was about five times the partner effect between these variables, the person’s own extradyadic stress seems more likely to spill over and increase the person’s intradyadic stress than the partner’s extradyadic stress.

In the present study, women and men did not significantly differ in their partner effects between extra- and intradyadic stress. This is in line with observations of Breitenstein et al. [103], showing that women and men seem to be equally affected by their partners’ extradyadic stress. However, some former studies reported that women would be more vulnerable to their partner’s stress [16] or vice versa [8]. Hence, there is no evidence for a clear direction. It can be assumed that women and men do not differ in their vulnerability to their partner’s stress in general but other confounding variables (e.g., personality traits, role distribution, internalized gender stereotypes) associated with sex could be responsible for sex differences found by other studies.

Contrary to our predictions and previous studies’ findings [8, 68, 69], neither the partner’s extradyadic stress 2 years after birth nor partner’s intradyadic stress 3 years after birth were directly associated with the person’s own depressive symptoms 3 years after birth in the APIMeM. Associations were only found in the bivariate correlation analyses. Therefore, any kind of stress experienced by the partner was not predictive for the person’s depressive symptoms when accounting for the person’s own stress. This suggests that the person’s own stress seems to be more decisive for their depressive symptoms than the partner’s stress. One possible explanation is the difference in cognitive and emotional demands of the person’s own vs. the partner’s stress. The associations between stress and depressive symptoms within a person can be explained because the person might be preoccupied with their own stressors, for instance, ruminating about them, which in turn is associated with depressive symptoms [104]. In reaction to the partner expressing their stress on the other hand, the person might show coping behavior [11] and provide support if their own resources are not exceeded [105]. Providing support can also be perceived as rewarding and evoke positive emotions [106]. Although we have chosen a population of parents of toddlers who are exposed to numerous extradyadic stressors such as parenting stress and the challenge of balancing family and work, the majority of our sample was healthy and mostly not distressed. Hence, it is possible that the person’s mental health was not impaired by their partner’s stress because their personal resources to deal with their partners’ stress and provide support were not exceeded. It would be promising to evaluate interaction effects between the partners’ stress to test this hypothesis.

Although there was no direct association between a partner’s extradyadic stress and the person’s own depressive symptoms, we found an indirect effect. The person’s own, but not the partner’s intradyadic stress mediated the association between the partner’s extradyadic stress and the person’s own depressive symptoms one year later. As there was no direct effect, we found a full mediation, indicating that the partner’s extradyadic stress affected the person’s own depressive symptoms mainly by increasing the person’s intradyadic stress. Our results suggest that the partner’s perception of extra- and intradyadic stress itself might not influence the person’s depressive symptoms. Instead, it might be the partner’s behavior in response to their extradyadic stress (e.g., little participation in chores, inattention, hostility, poor problem solving, or demanding too much support) that elicits intradyadic stress [43] and, in turn, depressive symptoms in the person. Supporting this assumption, there is evidence that a person’s depressive symptoms are increased by the partner’s hostile behavior towards the person [107] and an unequal distribution of chores [108].

However, the between-partner evidence for the reported extradyadic–intradyadic stress spillover and its potential influence on a person’s depressive symptoms needs further research because effect sizes were small and the partner’s extradyadic stress did not predict the person’s intradyadic stress beyond the former levels of intradyadic stress, as shown in the sensitivity analysis with autoregressive pathways. This could indicate a redundancy effect, i.e., the partner’s extradyadic stress and the person’s own as well as the partner’s intradyadic stress 2 years after birth shared much variance predicting the person’s intradyadic stress one year later. Based on the assessment of chronic extra- and intradyadic stress over the last 10 months, it is possible that extra- and intradyadic stress had already interacted. Diary studies reported same-day effects of extradyadic stress increasing negative relationship behaviors [38–41] that can in turn increase stress inside the relationship. Therefore, the partner’s chronic extradyadic stress measured 2 years after birth might have already spilled over. The perception of the relationship and the partners’ behavior towards each other seem to persist, resulting in both partners’ intradyadic stress predicting the person’s intradyadic stress one year later.

### Contribution to the state of research

Our study makes several important contributions to the existing literature. Firstly, we applied a longitudinal perspective on the chronic extradyadic–intradyadic stress spillover, while prior literature is limited to cross-sectional associations [8, 14–16, 59, 103] or diary studies with rather short investigation periods (up to 14 days) [38–41]. The present study provides evidence that the disturbing influence of chronic extradyadic stress spilling over into the couple relationship may persist for several months.

Secondly, findings on the between-partner effects regarding their association between stress and depressive symptoms are still mixed. Our results suggest that the partner’s extradyadic stress (see main analysis) and intradyadic stress (see sensitivity analysis) are more influential for the person’s intradyadic stress than for the person’s depressive symptoms. We assume that the partner’s behavior in response to their stress may influence the person’s intradyadic stress.

Thirdly, based on within-person findings [59], we examined the mediating role of intradyadic stress in the association between extradyadic stress and depressive symptoms within the person and between the partners. We found a partially mediated within-person association and a fully mediated between-partner association, although the evidence within the person was stronger than between the partners. Our results suggest that increasing intradyadic stress disturbing the couple relationship seems to be one mechanism for how extradyadic stress can increase depressive symptoms.

Finally, despite the generally higher reported levels for stress and depressive symptoms in women (in line with Falconier et al. [8]), women and men did not differ in their vulnerability to the partner’s stress. Different studies reported inconsistent findings regarding sex differences in vulnerability to the partner’s extradyadic stress, as discussed above. Individual (e.g., personality traits), intradyadic (e.g., relationship parameters, role distribution), and society factors (e.g., changing gender stereotypes) could be investigated to explain these mixed results.

### Strengths and limitations

The current study has several strengths worth mentioning. The prospective cohort study DREAM made a longitudinal and prospective evaluation of stress and depressive symptoms in couples possible. Using a standardized and validated questionnaire [77], a distinction between extra- and intradyadic stress was applied which is necessary, for example, due to different coping demands [17].

The sample size of 878 dyads provided high power to detect even small to medium sized effects and was larger than samples of related studies [8, 14–16, 46, 56, 69]. Furthermore, we investigated the stress spillover’s association with mental health in a previously neglected group: parents of toddlers (age two to three years).

In contrast to previous studies investigating parents with toddlers, we considered both mothers and fathers and their interdependence. For this purpose, the data were analyzed in a dyadic way using an advanced APIMeM. Due to the interdependence of romantic partners [7], it is beneficial to consider the partner’s potential influence evaluating a person’s mental health [61]. Moreover, the performed sensitivity analysis with autoregressive pathways enabled more meaningful statements about the direction of the associations and prevented the results from being confounded by the correlations in observations of the same variable over time [98, 99].

Nevertheless, the study also has some limitations. As the study had an observational design, the results cannot be interpreted in a causal manner, although autoregressive pathways were included.

The DREAM cohort examines a comprehensive picture of (transitioning to) parenthood regarding multiple facets (especially work, mental health, family relationships) over a long period of time. To minimize attrition by reducing participant burden, the timing and intervals between the measurement points, as well as the various constructs assessed, were chosen with particular care. For this reason, the constructs of interest were only available for the mediation analysis at two measurement points (T4 and T5) at the time of the current study.

Due to methodical restrictions, the sample comprised only parents who are in a relationship since pregnancy. As relationship stability is associated with extra- and intradyadic factors [109] as well as depressive symptoms [6], the exclusion criterion likely interferes with the primary variables of this study.

Given that the sample consisted of well-educated, little distressed parents of toddlers who are in a stable opposite-sex relationship, our results might not be applicable to couples in general. However, an above-average educational level compared to the local population [75, 76] is typical for participants in longitudinal family studies [74].

Extra- and intradyadic stress were retrospectively assessed over long time periods (ten or twelve months), which carries the risk for memory bias. Moreover, the chronic intradyadic stress of the last twelve months was assessed at the same measurement point as acute depressive symptoms of the last seven days. Therefore, it is possible that the acute depressive symptoms influenced the retrospective appraisal of chronic intradyadic stress and the proposed direction of the association may be less determinable. As there is evidence that retrospective assessments of stress and depressive symptoms are reliable, particularly in people of high socioeconomic status [110], we assume that our study nevertheless used data of good quality.

### Future research and practical implications

#### Recommendations for future studies and further research ideas

As findings on the potential influence of the partner’s stress on the person’s own depressive symptoms differed between main and sensitivity analyses, there is a need for replication studies. Specialized panel studies could be used to collect more detailed trends over time. This would make it possible to investigate mediation effects in an even more ideal way. Hereby, it would be worthwhile to look more closely at parenting stress, which was only one aspect of extradyadic stress considered in the current study, but which appears to be particularly important during this period of life.

Since similar studies investigated the dyadic association between stress and depressive symptoms mainly with opposite-sex parents in the prenatal period [46, 56, 69], it is necessary to address other populations (e.g., same-sex couples, childless couples, couples with adolescent children, couples who have one partner diagnosed with a mental health disorder) and a more heterogeneous sample to make statements about couples in general.

Moreover, future studies could extend the parameters used to evaluate the dyadic association between stress and mental health. It might be interesting to additionally evaluate partners’ interdependence regarding biological correlates to perceived stress like hair cortisol or to investigate the influence of the stress spillover on other domains of mental health (e.g., anxiety, sleep, anger-hostility, or substance use) besides depressive symptoms. Future studies could also examine other mediators besides intradyadic stress, such as relationship satisfaction [46], or include moderators, such as personality traits (e.g., neuroticism), role distribution, or dyadic coping [35] which is associated with mental health [111] and buffers the stress spillover [103] and the association between stress and mental health [71].

Finally, future studies could shift the perspective from the couple to the whole family. Since children are an integral part of the family with an important influence on the family climate and collective well-being, it would be promising to include children’s mental health (operationalized by observed behavior or older children’s self-report) and carry out triadic analyses to gain a deeper and more holistic understanding of the complex dynamics in families.

#### Practical implications

We found evidence that spillover seems to be one mechanism by which extradyadic stress can impair mental health even in a low stress population. Thus, it is important not only to consider stress spillover in psychotherapy, but also to develop preventive measures for parents in general. It could be beneficial to increase couples’ awareness of how stress originating outside the couple relationship can affect their behavior towards each other, their perception of the partner, and their relationship as a whole by developing educational material. This material could be used in preventive interventions like workshops for employees with highly stressful jobs, parenting workshops, couple counseling, or even couple therapy.

If the results can be replicated in a clinical sample, considering the partner’s stress and stress transmission in couples in therapy could lead to a more holistic approach and increase effectiveness. This could be done with dyadic interventions like those developed by Bodenmann et al. [112, 113] to improve stress management, communication in relationships as well as dyadic support and coping. Those interventions have proven to be effective in treatment of depressive symptoms [114].

## Conclusion

The present study was based on data of the prospective cohort study DREAM. We applied a dyadic perspective on the long-term association between stress and depressive symptoms by distinguishing between stress originating outside and inside the couple relationship. Our findings suggest that the person’s own and the partner’s stress from outside the couple relationship can spill over and increase the person’s stress inside the relationship. This spillover seems to negatively influence the person’s own mental health by increasing depressive symptoms. As our results regarding the influence of the partner’s stress differ between main and sensitivity analyses, further studies should investigate the stress spillover’s impact on mental health with dyadic methods including a more diverse sample, different mental health outcomes, and additional potential mediators and moderators.

## Supporting information

S1 Checklist STROBE statement—checklist of items that should be included in reports of observational studies. (DOCX)

S1 Table Saturated model. (PDF)

S2 Table Constrained model. (PDF)

S3 Table Model of main analysis. (PDF)

S4 Table Model of sensitivity analysis (incl. autoregression). (PDF)
