# Supplementary material for: The prospective impact of extradyadic stress on depressive symptoms and the mediating role of intradyadic stress in parents–an actor-partner interdependence mediation model
Source: PLoS One. 2024 Nov 5;19(11):e0311989. doi: 10.1371/journal.pone.0311989 (PMC11537395; doi:10.1371/journal.pone.0311989)
Supplement: S1 Checklist — (DOCX) [file pone.0311989.s001.docx]

STROBE Statement—checklist of items that should be included in reports of observational studies

|  | Item No. | Recommendation | Page  No. | Relevant text from manuscript |
| --- | --- | --- | --- | --- |
| **Title and abstract** | 1 | (*a*) Indicate the study’s design with a commonly used term in the title or the abstract | 1,2 | **Title:** “The prospective impact of extradyadic stress on depressive symptoms and the mediating role of intradyadic stress in parents – an actor-partner interdependence mediation model”  **Abstract:** “Longitudinal data of a community sample of 878 opposite-sex couples, participating in the prospective cohort study DREAM, were collected two and three years after birth.” |
|  |  | (*b*) Provide in the abstract an informative and balanced summary of what was done and what was found | 2 | **Abstract** |
| Introduction | | | |  |
| Background/rationale | 2 | Explain the scientific background and rationale for the investigation being reported | 3 – 7 | **Introduction** |
| Objectives | 3 | State specific objectives, including any prespecified hypotheses | 6 – 7 | **Introduction – Current study** Objectives (ll. 194 – 202) and  Hypotheses (ll. 222 – 230) included |
| Methods | | | |  |
| Study design | 4 | Present key elements of study design early in the paper | 7 – 8 | **Material and Methods – Design** (ll. 233 – 237) |
| Setting | 5 | Describe the setting, locations, and relevant dates, including periods of recruitment, exposure, follow-up, and data collection | 8 | **Material and Methods – Design** (ll. 239 – 251) |
| Participants | 6 | (*a*) *Cohort study*—Give the eligibility criteria, and the sources and methods of selection of participants. Describe methods of follow-up  *Case-control study*—Give the eligibility criteria, and the sources and methods of case ascertainment and control selection. Give the rationale for the choice of cases and controls  *Cross-sectional study*—Give the eligibility criteria, and the sources and methods of selection of participants | 8  8 | **Material and Methods – Design** (ll. 237 – 239)  **Material and Methods – Sample** (ll. 254 – 268) |
|  |  | (*b*) *Cohort study*—For matched studies, give matching criteria and number of exposed and unexposed  *Case-control study*—For matched studies, give matching criteria and the number of controls per case |  |  |
| Variables | 7 | Clearly define all outcomes, exposures, predictors, potential confounders, and effect modifiers. Give diagnostic criteria, if applicable | 9 – 10 | **Material and Methods – Measures** |
| Data sources/ measurement | 8* | For each variable of interest, give sources of data and details of methods of assessment (measurement). Describe comparability of assessment methods if there is more than one group | 9 – 10 | **Material and Methods – Measures** |
| Bias | 9 | Describe any efforts to address potential sources of bias | 10  11  14 – 15  20 – 21  23 | **Material and Methods – Measures** (ll. 325 – 336)  **Material and Methods – Data analyses** (ll. 373 – 380)  **Results – Attrition analyses**  **Discussion – Stress and depressive symptoms – between-partner effects**  (ll. 644 – 648)  **Discussion – Strengths and limitation** (ll. 717 – 735) |
| Study size | 10 | Explain how the study size was arrived at | 11 – 12 | **Material and Methods – Sample**, including “Fig 2. Flowchart of retention rates, attrition, and exclusion criteria” |

Continued on next page

| Quantitative variables | 11 | Explain how quantitative variables were handled in the analyses. If applicable, describe which groupings were chosen and why | 9 – 10  10 – 11 | **Material and Methods – Measures**  **Material and Methods – Data analyses** (ll. 337 – 380) |
| --- | --- | --- | --- | --- |
| Statistical methods | 12 | (*a*) Describe all statistical methods, including those used to control for confounding | 10 – 12 | **Material and Methods – Data analyses** |
|  |  | (*b*) Describe any methods used to examine subgroups and interactions | 11 | **Material and Methods – Data analyses** (ll. 364 – 368) |
|  |  | (*c*) Explain how missing data were addressed | 8  9  11 | **Material and Methods – Sample**  (ll. 267 – 268)  **Material and Methods – Measures** (ll. 282 – 283)  **Material and Methods – Data analyses** (ll. 350 – 351) |
|  |  | (*d*) *Cohort study*—If applicable, explain how loss to follow-up was addressed  *Case-control study*—If applicable, explain how matching of cases and controls was addressed  *Cross-sectional study*—If applicable, describe analytical methods taking account of sampling strategy | 8  14 – 15 | **Material and Methods – Sample**  (ll. 267 – 268)  **Results – Attrition analyses** |
|  |  | (*e*) Describe any sensitivity analyses | 11 | **Material and Methods – Data analyses** (ll. 373 – 380) |
| Results | | | | |
| Participants | 13* | (a) Report numbers of individuals at each stage of study—eg numbers potentially eligible, examined for eligibility, confirmed eligible, included in the study, completing follow-up, and analysed | 8 – 9 | **Material and Methods – Sample**, including “Fig 2. Flowchart of retention rates, attrition, and exclusion criteria” |
|  |  | (b) Give reasons for non-participation at each stage | 8 – 9 | **Material and Methods – Sample**, including “Fig 2. Flowchart of retention rates, attrition, and exclusion criteria” |
|  |  | (c) Consider use of a flow diagram | 9 | **Material and Methods – Sample**  “Fig 2. Flowchart of retention rates, attrition, and exclusion criteria” |
| Descriptive data | 14* | (a) Give characteristics of study participants (eg demographic, clinical, social) and information on exposures and potential confounders | 12 – 13 | **Results – Sample characteristics,** including “Table 1. Sample characteristics” |
|  |  | (b) Indicate number of participants with missing data for each variable of interest | 12 – 13 | **Results – Sample characteristics,** “Table 1. Sample characteristics” |
|  |  | (c) *Cohort study*—Summarise follow-up time (eg, average and total amount) | 8 | **Material and Methods – Design**  (ll. 242 – 251) |
| Outcome data | 15* | *Cohort study*—Report numbers of outcome events or summary measures over time | 13 – 19 | **Results** |
|  |  | *Case-control study—*Report numbers in each exposure category, or summary measures of exposure |  |  |
|  |  | *Cross-sectional study—*Report numbers of outcome events or summary measures |  |  |
| Main results | 16 | (*a*) Give unadjusted estimates and, if applicable, confounder-adjusted estimates and their precision (eg, 95% confidence interval). Make clear which confounders were adjusted for and why they were included | 16 – 19  33 – 38 | **Results – Main analyses,** including “Fig 3. Standardized coefficients (∆) of direct effects in the actor-partner interdependence mediation model (APIMeM) with included confounder (academic degree)” and “Table 3. Indirect effects in the actor-partner interdependence mediation model (APIMeM) with included confounder (academic degree”  **Supplementary material** |
|  |  | (*b*) Report category boundaries when continuous variables were categorized | 9 – 10 | **Material and Methods – Measures** |
|  |  | (*c*) If relevant, consider translating estimates of relative risk into absolute risk for a meaningful time period |  |  |

Continued on next page

| Other analyses | 17 | Report other analyses done—eg analyses of subgroups and interactions, and sensitivity analyses | 15 – 16  16  18 – 19 | **Results – Correlational analyses**  **Results – Main analyses – Model selection**  **Results – Main analyses – Results when controlling for autoregression** |
| --- | --- | --- | --- | --- |
| Discussion | | | | |
| Key results | 18 | Summarise key results with reference to study objectives | 19 | **Discussion** (ll. 575 – 586) |
| Limitations | 19 | Discuss limitations of the study, taking into account sources of potential bias or imprecision. Discuss both direction and magnitude of any potential bias | 23 | **Discussion – Strengths and limitations** (ll. 717 – 735) |
| Interpretation | 20 | Give a cautious overall interpretation of results considering objectives, limitations, multiplicity of analyses, results from similar studies, and other relevant evidence | 19 – 24 | **Discussion** |
| Generalisability | 21 | Discuss the generalisability (external validity) of the study results | 23  23 | **Discussion – Strengths and limitation** (ll. 724 – 727)  **Discussion – Future research and practical implication** (ll. 740 – 744) |
| Other information | |  | | |
| Funding | 22 | Give the source of funding and the role of the funders for the present study and, if applicable, for the original study on which the present article is based | 25 | **Funding information**  “The DREAM study is funded by the Deutsche Forschungsgemeinschaft (DFG, German Research Foundation) [grant numbers GA 2287/4-1 and GA 2287/4-2]. Susan Garthus-Niegel is a management committee member of COST action CA18211.” (ll. 792 – 796) |

*Give information separately for cases and controls in case-control studies and, if applicable, for exposed and unexposed groups in cohort and cross-sectional studies.

**Note:** An Explanation and Elaboration article discusses each checklist item and gives methodological background and published examples of transparent reporting. The STROBE checklist is best used in conjunction with this article (freely available on the Web sites of PLoS Medicine at http://www.plosmedicine.org/, Annals of Internal Medicine at http://www.annals.org/, and Epidemiology at http://www.epidem.com/). Information on the STROBE Initiative is available at www.strobe-statement.org.
