# Supplementary material for: The prospective impact of extradyadic stress on depressive symptoms and the mediating role of intradyadic stress in parents–an actor-partner interdependence mediation model
Source: PLoS One. 2024 Nov 5;19(11):e0311989. doi: 10.1371/journal.pone.0311989 (PMC11537395; doi:10.1371/journal.pone.0311989)
Supplement: S1 Table — (PDF) [file pone.0311989.s002.pdf]

S1 Table. Saturated model

Saturated actor-partner interdependence mediation model (APIMeM) for testing the mediating effect of intradyadic stress (IS) in the association between extradyadic stress (ES) and depressive symptoms (DS).

	Women						Men					
	b	**Δ**	***SE***	***p***	**95 %-CI**		***b***	**Δ**	***SE***	***p***	**95 %-CI**	
					**Lower**	**Upper**					**Lower**	**Upper**
**Direct actor effects**												
$\mathbf{ES}_A \rightarrow \mathbf{IS}_A$	0.456	0.397	0.050	**<.001**	0.358	0.551	0.451	0.408	0.054	**<.001**	0.348	0.559
$\mathbf{IS}_A \rightarrow \mathbf{DS}_A$	2.596	0.280	0.507	**<.001**	1.603	3.574	2.096	0.231	0.433	**<.001**	1.265	2.984
$\mathbf{ES}_A \rightarrow \mathbf{DS}_A$	3.562	0.335	0.490	**<.001**	2.566	4.494	3.557	0.354	0.508	**<.001**	2.572	4.558
**Direct partner effects**												
$\mathbf{ES}_P \rightarrow \mathbf{IS}_A$	0.116	0.091	0.058	**.046**	0.002	0.230	0.064	0.064	0.041	.121	−0.015	0.148
$\mathbf{IS}_P \rightarrow \mathbf{DS}_A$	0.230	0.022	0.597	.700	−0.948	1.412	0.304	0.038	0.401	.448	−0.491	1.096
$\mathbf{ES}_P \rightarrow \mathbf{DS}_A$	−0.042	−0.004	0.591	.943	−1.188	1.143	−0.330	−0.036	0.443	.456	−1.186	0.550
**Specific indirect effects**												
$\mathbf{ES}_A \rightarrow \mathbf{IS}_A \rightarrow \mathbf{DS}_A$	1.183	0.111	0.272	**<.001**	0.672	1.752	0.946	0.094	0.221	**<.001**	0.550	1.405
$\mathbf{ES}_P \rightarrow \mathbf{IS}_A \rightarrow \mathbf{DS}_A$	0.302	0.026	0.159	.057	0.006	0.633	0.134	0.015	0.095	.158	−0.031	0.346
$\mathbf{ES}_P \rightarrow \mathbf{IS}_P \rightarrow \mathbf{DS}_A$	0.104	0.009	0.273	.704	−0.422	0.655	0.139	0.015	0.184	.451	−0.221	0.498
$\mathbf{ES}_A \rightarrow \mathbf{IS}_P \rightarrow \mathbf{DS}_A$	0.015	0.001	0.048	.761	−0.076	0.131	0.035	0.003	0.054	.513	−0.064	0.156

b = unstandardized coefficients. Δ = standardized coefficients separated by sex. *SE* = standard errors of *b*. Two-tailed *p*-values ($p < .05$ in bold). Bootstrapped 95%-CIs (5,000 iterations). A = actor. P = partner.

χ^2 = 0.000 ($df = 0$, $p = .000$). RMSEA = 0.000. CFI = 1.000. TLI = 1.000.
