# Supplementary material for: The prospective impact of extradyadic stress on depressive symptoms and the mediating role of intradyadic stress in parents–an actor-partner interdependence mediation model
Source: PLoS One. 2024 Nov 5;19(11):e0311989. doi: 10.1371/journal.pone.0311989 (PMC11537395; doi:10.1371/journal.pone.0311989)
Supplement: S2 Table — (PDF) [file pone.0311989.s003.pdf]

S2 Table. Constrained model

Constrained actor-partner interdependence mediation model (APIMeM) for testing the mediating effect of intradyadic stress (IS) in the association between extradyadic stress (ES) and depressive symptoms (DS).

	b	$\Delta_{♀/♂}$	*SE*	*p*	95 %-CI	
					**Lower**	**Upper**
**Direct actor effects**						
$\mathbf{ES_A \rightarrow IS_A}$	0.453	0.395/0.410	0.034	<**.001**	0.389	0.519
$\mathbf{IS_A \rightarrow DS_A}$	2.369	0.255/0.261	0.347	<**.001**	1.689	3.053
$\mathbf{ES_A \rightarrow DS_A}$	3.552	0.334/0.353	0.355	<**.001**	2.859	4.252
**Direct partner effects**						
$\mathbf{ES_P \rightarrow IS_A}$	0.084	0.066/0.084	0.031	**.007**	0.023	0.146
$\mathbf{IS_P \rightarrow DS_A}$	0.257	0.024/0.033	0.350	.462	−0.414	0.959
$\mathbf{ES_P \rightarrow DS_A}$	−0.209	−0.018/−0.023	0.361	.562	−0.914	0.502
**Specific indirect effects**						
$\mathbf{ES_A \rightarrow IS_A \rightarrow DS_A}$	1.073	0.101/0.107	0.175	<**.001**	0.751	1.433
$\mathbf{ES_P \rightarrow IS_A \rightarrow DS_A}$	0.200	0.017/0.022	0.079	**.011**	0.053	0.367
$\mathbf{ES_P \rightarrow IS_P \rightarrow DS_A}$	0.116	0.010/0.013	0.159	.464	−0.189	0.433
$\mathbf{ES_A \rightarrow IS_P \rightarrow DS_A}$	0.022	0.002/0.002	0.033	.511	−0.037	0.095

b = unstandardized coefficients. Δ = standardized coefficients separated by sex. *SE* = standard errors of *b*. Two-tailed *p*-values ($p < .05$ in bold). Bootstrapped 95%-CIs (5,000 iterations). A = actor. P = partner.

$\chi^2 = 2.273$ ($df = 6$, $p = .893$). RMSEA = 0.000. CFI = 1.000. TLI = 1.000.
