# Supplementary material for: The prospective impact of extradyadic stress on depressive symptoms and the mediating role of intradyadic stress in parents–an actor-partner interdependence mediation model
Source: PLoS One. 2024 Nov 5;19(11):e0311989. doi: 10.1371/journal.pone.0311989 (PMC11537395; doi:10.1371/journal.pone.0311989)
Supplement: S3 Table — (PDF) [file pone.0311989.s004.pdf]

S3 Table. Model of main analysis

Partially constrained actor-partner interdependence mediation model (APIMeM) for testing the mediating effect of intradyadic stress (IS) in the association between extradyadic stress (ES) and depressive symptoms (DS) with confounder academic degree (AD).

	$b_{♀/♂}$	$\Delta_{♀/♂}$	$SE_{♀/♂}$	$p_{♀/♂}$	**95 %-CI$_{♀/♂}$**	
					**Lower**	**Upper**
**Direct actor effects**						
**ES$_A$ → IS$_A$**	0.453	0.395/0.410	0.033	<**.001**	0.389	0.519
**IS$_A$ → DS$_A$**	2.338	0.252/0.257	0.346	<**.001**	1.660	3.017
**ES$_A$ → DS$_A$**	3.582	0.336/0.356	0.355	<**.001**	2.901	4.282
**Direct partner effects**						
**ES$_P$ → IS$_A$**	0.085	0.067/0.085	0.031	**.007**	0.022	0.147
**IS$_P$ → DS$_A$**	0.245	0.023/0.031	0.348	.481	−0.424	0.930
**ES$_P$ → DS$_A$**	−0.196	−0.017/−0.022	0.360	.587	−0.893	0.512
**Confounding influence**						
**AD$_A$ → IS$_A$**	−0.063/−0.017	—[a]	0.040/0.038	.117/.663	−0.143/−0.090	0.015/0.058
**AD$_A$ → DS$_A$**	−0.912/0.275	—[a]	0.390/0.357	**.019**/.442	−1.679/−0.431	−0.152/0.963
**Specific indirect effects**						
**ES$_A$ → IS$_A$ → DS$_A$**	1.060	0.100/0.105	0.175	<**.001**	0.736	1.419
**ES$_P$ → IS$_A$ → DS$_A$**	0.198	0.017/0.022	0.078	**.011**	0.050	0.364
**ES$_P$ → IS$_P$ → DS$_A$**	0.111	0.009/0.012	0.158	.483	−0.198	0.427
**ES$_A$ → IS$_P$ → DS$_A$**	0.021	0.002/0.002	0.033	.530	−0.038	0.095

b = unstandardized coefficients. Δ = standardized coefficients separated by sex. *SE* = standard errors of *b*. Two-tailed *p*-values ($p < .05$ in bold). Bootstrapped 95%-CIs (5,000 iterations). A = actor. P = partner.

χ^2 = 8.948 (df = 10, p = .537). RMSEA = 0.000. CFI = 1.000. TLI = 1.000.

[a] A report of standardized coefficients (Δ) of direct effects of academic degree was not possible as academic degree was a dichotomous variable.
