# Supplementary material for: The prospective impact of extradyadic stress on depressive symptoms and the mediating role of intradyadic stress in parents–an actor-partner interdependence mediation model
Source: PLoS One. 2024 Nov 5;19(11):e0311989. doi: 10.1371/journal.pone.0311989 (PMC11537395; doi:10.1371/journal.pone.0311989)
Supplement: S4 Table — (PDF) [file pone.0311989.s005.pdf]

S4 Table. Model of sensitivity analysis (incl. autoregression)

Partially constrained actor-partner interdependence mediation model (APIMeM) for testing the mediating effect of intradyadic stress (IS) in the association between extradyadic stress (ES) and depressive symptoms (DS) with confounder academic degree (AD) and autoregression.

	$b_{♀/♂}$	$Δ_{♀/♂}$	$SE_{♀/♂}$	$p_{♀/♂}$	95 %-CI♀/♂	
					**Lower**	**Upper**
**Direct actor effects**						
**ES_A (T4) → IS_A (T5)**	0.088	0.077/0.080	0.032	**.005**	0.029	0.150
**IS_A (T5) → DS_A (T5)**	1.573	0.170/0.173	0.311	<**.001**	0.973	2.179
**ES_A (T4) → DS_A (T5)**	1.740	0.163/0.173	0.372	<**.001**	1.015	2.473
**IS_A (T4) → IS_A (T5)**	0.683	0.685/0.676	0.033	<**.001**	0.618	0.747
**DS_A (T4) → DS_A (T5)**	0.465	0.445/0.430	0.036	<**.001**	0.395	0.536
**Direct partner effects**						
**ES_P (T4) → IS_A (T5)**	−0.031	−0.024/−0.031	0.028	.265	−0.085	0.024
**IS_P (T5) → DS_A (T5)**	0.286	0.027/0.036	0.307	.353	−0.323	0.890
**ES_P (T4) → DS_A (T5)**	−0.315	−0.027/−0.035	0.340	.354	−0.985	0.345
**IS_P (T4) → IS_A (T5)**	0.060	0.052/0.069	0.029	**.035**	0.002	0.116
**DS_P (T4) → DS_A (T5)**	−0.042	−0.033/−0.047	0.032	.183	−0.104	0.021
**Confounding influence of AD**						
**AD_A → IS_A (T5)**	−0.012/0.012	—[a]	0.032/0.029	.702/.670	−0.075/−0.043	0.050/0.069
**AD_A → DS_A (T5)**	−0.654/0.187	—[a]	0.352/0.317	.063/.555	−1.340/−0.434	0.034/0.799
**Specific indirect effects**						
**ES_A (T4) → IS_A (T5) → DS_A (T5)**	0.139	0.013/0.014	0.058	**.017**	0.041	0.270
**ES_P (T4) → IS_A (T5) → DS_A (T5)**	−0.049	−0.004/−0.005	0.046	.288	−0.141	0.038
**ES_P (T4) → IS_P (T5) → DS_A (T5)**	0.025	0.002/0.003	0.031	.409	−0.028	0.095
**ES_A (T4) → IS_P (T5) → DS_A (T5)**	−0.009	−0.001/−0.001	0.015	.561	−0.047	0.014

b = unstandardized coefficients. Δ = standardized coefficients separated by sex. *SE* = standard errors of *b*. Two-tailed *p*-values ($p < .05$ in bold). Bootstrapped 95%-CIs (5,000 iterations). A = actor. P = partner. T4 = 2 years after birth. T5 = 3 years after birth.

$\chi^2 = 26.432$ ($df = 22$, $p = .234$). RMSEA = 0.015. CFI = .997. TLI = .994.

[a] A report of standardized coefficients (Δ) of direct effects of academic degree was not possible as academic degree was a dichotomous variable.
